# The Use of New Waste-Based Plasticizer Made from Modified Used Palm Oil for Non-Glutinous Thermoplastic Starch Foam

**DOI:** 10.3390/polym14193997

**Published:** 2022-09-24

**Authors:** Jatupol Junthip, Natchapat Chaipalee, Yada Sangsorn, Chanannat Maspornpat, Juthamas Jitcharoen, Sittichai Limrungruengrat, Thana Chotchuangchutchaval, Ekkachai Martwong, Nathapong Sukhawipat

**Affiliations:** 1 Faculty of Science and Technology, Nakhon Ratchasima Rajabhat University, Nakhon Ratchasima 30000, Thailand; 2 Department of Mechanical Engineering Technology, College of Industrial Technology, King Mongkut’s University of Technology North Bangkok, Bangkok 10800, Thailand; 3 Department of Chemistry, Faculty of Science, Ubon Ratchathani University, Ubon Ratchathani 34190, Thailand; 4 Center of Sustainable and Energy Engineering Materials, Department of Mechanical Engineering Technology, College of Industrial Technology, King Mongkut’s University of Technology North Bangkok, Bangkok 10800, Thailand; 5 Division of Science, Faculty of Science and Technology, Rajamangala University of Technology Suvarnabhumi, Phra Nakhon Si Ayutthaya, 13000, Thailand

**Keywords:** thermoplastic starch foam, non-glutinous starch, used palm oil, green plasticizer, properties improvement

## Abstract

A novel waste-based plasticizer derived from modified used palm oil (mUPO) was successfully developed and has been used as a new plasticizer to non-glutinous thermoplastic starch foam to improve their properties. The molecular weight and hydroxyl number of the mUPO was 3150 g/mol and 192.19 mgOH/g, respectively. The effects of mUPO content ranging from 0 to 9 phr were investigated. The results revealed that the optimal mUPO content as an additive was 6 wt%. The addition of mUPO had a direct effect on the mechanical properties and thermal properties. The impact strength increased from 1.30 to 4.55 J/m, while the glass transition temperature (*T_g_*) decreased from 70.83 to 66.50 °C by increasing mUPO from 0 phr to 6 phr in the thermoplastic starch foam. The mUPO, on the other hand, has also the potential to reduce shrinkage from 33.91 to 21.77% and moisture absorption from 5.93 to 1.73% by increasing the content from 0 phr to 6 phr in starch foam. Furthermore, the mUPO helps the forming of the foam structure as measured by SEM, and the mUPO utilization of waste-based material could be a promising green alternative plasticizer for starch components, especially starch foam applications.

## 1. Introduction

Polymer-based foams are now widely used in our daily life in applications such as medical engineering, packaging, sound absorption, and insulation due to their light weight, high mechanical properties, portability, and ease of processing and control [1]. Almost all of them are made of petroleum-based polymers, such as styrene foam for food packaging and polyurethane foam for heat insulation and sound absorption. Petroleum-based foam brings concerns to environmental issues such as greenhouse effects and air pollution, especially in one-time-use packaging applications. To avoid these drawbacks, many research groups are attempting to develop new green foam made from biopolymer-based polylactic acid [2], polybutylene succinate [3], and starch foam, particularly one-time-use material.

There are numerous bio-based starches that have been developed and used to prepare foam material, including potato starch [2,4,5,6,7,8,9], corn starch [10,11], cassava starch [12,13,14], wheat starch [15], and others. The different starch sources were used, and the different properties of thermoplastic starch foam were discovered. Rice starch, produced from broken rice, is one of the interesting resources to use as a substrate for the foam matrix in this work. According to Machado et al. [16], rice starch can help improve the mechanical, water absorption, and thermal properties of cassava starch-based foam. According to Williams et al. [17], the high content of amylose in rice-based starch is the key to improving the properties of starch-based materials. Even though the properties of rice-based starch are good, there are no reports of neat rice starch-based foam. They were used as an additive in other starch-based foams. As a preliminary study, this work is interested in preparing thermoplastic starch foam based on rice starch in the class of non-glutinous starch foam.

Despite the fact that rice starch is a bio-based resource and an environmentally friendly material, starch is brittle and water sensitive on its own. As a result, a proper plasticizer is incorporated to the starch matrix to improve flexibility and water sensitivity. Plasticizers have a low molecular weight and form secondary bonds with polymer chains to spread them apart. Thus, plasticizers increase chain mobility while decreasing secondary polymer–polymer bonding, resulting in materials with a softer mass and lower modulus [18]. The most commonly used plasticizer for starch is petroleum-based glycerol. In 2005, Mali et al. [19] used glycerol to plasticize different starches. The findings revealed that its thermal, mechanical, and barrier properties had improved. In 2014, Silva et al. [20] reported the use of glycerol, sorbitol, and polyols as thermoplastic starch plasticizers. They stated that efficient plasticization is enabled by the short, flexible, and hydrogen-bonding dangling side chains that are connected to the core of the plasticizer, leaving crystalline starch domains to allow self-reinforcement. Sanyan et al. [21] used glycerol and sorbitol as plasticizers in sugar palm starch in 2015. The effect of plasticizer concentration on the thermal properties of plasticized thermoplastic starch was insignificant. However, at a higher plasticizer concentration (45 wt%), it was decreased due to the anti-plasticization effect of plasticizers. Bergel et al. [5] investigated the effects of a glycerol plasticizer on corn starch, potato starch, and cassava starch in 2017. When compared to other foams, foams made from starch, glycerol, and water in the weight proportion of 62/5/33 were denser and had a higher modulus. In 2020, Kahvand and Fasihi [22] investigated the microstructure and thermal properties of thermoplastic starch foam. The morphology revealed that increasing the plasticizer content increased the thermal properties while decreasing the cell size of the thermoplastic starch foam. In the same year, Machado et al. [12] investigated the effects of glycerol on cassava starch-blended acetate–cassava starch foam. It was discovered that 13 wt% glycerol had the best properties of the blend starch foam. In 2021, Tarique et al. [23] improved the physical, mechanical, thermal, and barrier properties of arrowroot (*Maranta arundinacea*) starch biopolymers by loading glycerol into the matrix. According to the literature reviews, the addition of plasticizer allows mixing and interacting with the starch, resulting in a significant improvement in starch processability as a thermoplastic and their properties, particularly thermal and mechanical properties. According to the report of Silva et al. [20], petroleum-based polyols are also used as an effective plasticizer for thermoplastic starch. Hence, in this work, the bio-based polyol made from used palm oil is very interesting to replace the petroleum-based plasticizer. The goal of this study is to discover a new green plasticizer for thermoplastic starch foam in order to pave the way for the use of a waste-based material in the starch foam industrial sector.

Used palm oil (UPO) is a byproduct of the food preparation process, specifically fried food. According to the report of Prasertsan [24], there is a lot of waste made of used palm oil from Thai households every day. It has also been reported that approximately 1.2 million tons of wastewater per year were discovered due to the contamination of used palm oil waste in that source of water. To avoid the contamination of UPO waste in the environment, the best way to use UPO is to apply it to the targeted industrial sector. From the view of this point, the advantages of UPO include not only reduced environmental waste, but also waste value-added [25,26]. The method used in this work to apply UPO in the industrial sector is to modify the UPO as polyols with hydroxyl active groups as a plasticizer of starch foam.

Therefore, the purpose of this research is to investigate the potential of mUPO-based polyols as a plasticizer of non-glutinous thermoplastic starch foam. The effects of mUPO content were varied from 0 to 5 phr. The shrinkage, moisture absorption, thermal properties, mechanical properties, and morphology of thermoplastic starch foam were examined.

## 2. Materials and Methods

### 2.1. Materials

Non-glutinous rice flour (Erawan brand) was purchased from Do Heng Rice Vermicelli Factory Co., Ltd. (Nakhon Prathom, Thailand). Baking powder double-acting formula, containing 30% of sodium bicarbonate (NaHCO_3_), 24% of disodium pyrophosphate (Na_2_H_2_P_2_O_7_) and 16% of monocalcium phosphate (Ca(H_2_PO_4_)_2_), was purchased from Best Food brand (Chachoengsao, Thailand). Polyvinyl alcohol (PVOH), a moisture resistance, with a molecular weight of 72,000 g/mol was purchased from Krungthepchemi Co., Ltd. (Bangkok, Thailand). Used palm oil for preparing recycled palm oil (RPO) was purchased from a Nonthaburi local market, Nonthaburi province (acid value of 1.41 mgKOH/g and iodine value of 40.1 mg I_2_/g). The used palm oil was first filtered before modification. Hydrogen peroxide (H_2_O_2_, 35%) was purchased from Ajax Finechem (Sydney, Australia). Formic acid (HCOOH, 98%) was purchased from Fisher Chemical (Shanghai, China). Ethyl acetate (CH_3_COOC_2_H_5_, >99%) was purchased from RCI Lab-Scan Limited (Bangkok, Thailand). Distilled water, purchased from Yod Nam Distilled Water (Nakhon Prathom, Thailand), was used to prepare gelatinized starch before foam preparation. All ingredients were dried before use.

#### 2.1.1. Preparation of Modified Used Palm Oil (mUPO)-Based Plasticizer

RPO-based polyol was prepared from used pal oil (UPO). The process was set in a single step with both epoxidation and ring-opening reactions. Some 250 g (0.3 mol) of used palm oil was filtered and dried at 70 °C for 8 h before setting the reaction. Then, 57.71 mL (1.5 mol) of 98% formic acid was added dropwise in a 2 L reactor containing the UPO, followed by 50.25 mL (0.75 mol) of 35% hydrogen peroxide. The mixture was continuously stirred for 4 h with a controlled speed of 200 rpm at 70 °C. Then, the RPO was washed using ethyl acetate, saturated NaHCO_3_, and NaCl solutions. The mUPO was evaporated under vacuum in a rotary evaporator at 40 °C to obtain pure UPO-based plasticizer. The mUPO was further analyzed for its iodine value, acid value, and hydroxyl value.

The chemical structure confirmation of UPO and mUPO has been reported in our previous work [27,28,29,30]. The figure of ^1^H-NMR spectra and FTIR spectra is presented in Figures S1 and S2, respectively. The main structure is triglyceride structure, and the results are summarized as below.

***UPO***, ^1^H-NMR (400 MHz, CDCl_3_, δ): (1) 4.2 (t, 2H, –CH_2_O(C=O), (2) 5.1 (d, 1H, –CHO(C=O), (7) 5.3 (d, 1H, –CH = CH–)

FTIR spectroscopy (ν): 1150 (s, C-O-C), 1600 (s, C=O), 2800–2950 (s, C-H), 3100 (w, =CH).

***mUPO***, ^1^H-NMR (400 MHz, CDCl_3_, δ): (1) 4.2 (t, 2H, –CH_2_O(C=O), (2) 5.1 (d, 1H, –CHO(C=O), (11) 3.0 (d, 1H, –CH_2_CH(OH))

FTIR spectroscopy (ν): 1150 (s, C-O-C), 1600 (s, C=O), 2800–2950 (s, C-H), 3100 (w, =CH), 3300–3500 (s, OH).

#### 2.1.2. Preparation of Non-Glutinous Starch Foam

Polyvinyl alcohol (PVOH), a moisture resistance for starch foam, was dissolved in distilled water at a concentration of 4 wt%. It was started at room temperature with a concentration of 50 wt% non-glutinous starch dissolved in 4 wt% PVOH solution, by controlling the starch concentration of 92.5 wt% and the PVOH concentration of 7.5 wt% according to Boonchaisuriya and Chungsiriporn [31]. The total solid dried content of non-glutinous starch foam composite was set at 15 g. The component was thoroughly mixed at room temperature before adding baking powder at a concentration of 7.5 phr. The amount of mUPO was then well-mixed and varied between 0 and 4 phr by adding into the system prior to processing at high temperatures. The mixture was then stirred with a magnetic stirrer with a speed of 200 rpm at 60 °C until the gelatinized starch was evenly distributed. Finally, the homogeneous component was poured into a Teflon mold with sizes of 10 × 10 × 3 cm^3^ and dried at 180 °C for 45 min in the oven air. The sample was then collected and stored in the desiccator for 24 h before any testing. The formulation of non-glutinous starch foam was demonstrated in Table 1.

### 2.2. Characterizations

The iodine values of mUPO were determined by titration to assess the double bonds in structures, according to ISO3961-2009. The acid values were titrated to analyze the free fatty acids in the oils, according to ISO660-2009. The OH values were titrated to determine the number of OH units in the structures, according to ISO14900-2001.

The chemical structures of UPO and mUPO were further analyzed by ^1^H-NMR with a Bruker 400 Fourier transform spectrometer at 400.13 and 100.62 MHz. All samples were dissolved in CDCl_3_, using tetramethylsilane (TMS) as the internal standard. Fourier Transform Infrared (FTIR) spectra were recorded with a Nicolet Avatar 370 DTGS FTIR spectrometer over the range 4000–400 cm^−1^.

The molecular weights of the UPO and mUPO samples were analyzed by size exclusion chromatography (SEC) using a Shodex GPC KF-806M column (Tokyo, Japan).

Shrinkage percentage of starch foam composite was evaluated by measuring the volume of the sample before and after oven-drying at 180 °C for 45 min. The shrinkage percentage was calculated by the following Equation (1).

S (%) = [(V_f_ − V_i_)/V_i_] × 100 (1)

when: S (%) the shrinkage percentage of starch foam, V_i_ is the volume of the sample before drying (cm^3^), and V_f_ is the volume after drying (cm^3^).

The moisture absorption of the sample was studied by placing the sample into the desiccator with a control of 60% relative humidity. It was determined in five specimens for each formula. The sample was weighed every day. The moisture absorption rate was calculated by the following Equation (2).

A (%) = [(W_f_ − W_i_)/W_i_] × 100 (2)

when: A (%) is absorption percentage, W_i_ is the initial weight of the sample, and W_f_ is the weight after obtaining the moisture of the sample.

The mechanical properties of the sample were evaluated by flexural strength result via three-point bending using Testometric (M500-25AT) (Testometric, Rochdale, UK) on a universal testing machine in accordance with ASTM D790, with a crosshead speed of 1 mm/s at a span distance of 25 mm. The specimen sizes were 100 × 25 × 3 mm^3^. Three specimens were tested for one formulation, and the average value was reported.

Notched Izod impact testing was performed according to ASTM D256 in a CEAST^®^ series 6967 (Massachusetts, USA) impact-testing machine using a 2.75 J pendulum. The impact test specimens were 60 × 12 × 3 mm^3^. Three specimens were tested for one formulation and the average value was reported.

The morphology of the thermoplastic non-glutinous starch was examined using a scanning electron Mmicroscope (SEM) equipped with a QUANTA 450 (Thermo Fisher Scientific, Waltham, Massachusetts, USA) under high-vacuum and high-voltage settings at 20.00 kV. Before imaging, all of the samples were gold-coated. In addition to cross-checking the structure of the starch foam composite, an optical microscope with a magnification of 5× and Xenon (DN-117MS) (Nanjing Jiangnan Novel Optics, Nanjing, China) was utilized.

The thermal properties of starch foam composites were also tested. Differential Scanning Calorimetry (DSC) was carried on TGA/DSC3+ by METTLER TOLEDO (Bangkok, Thailand). The aluminum pan was loaded with a starting sample weight of about 10 mg, and the run was under nitrogen atmosphere (nitrogen flow = 100 mL.min^−1^) with a heating rate of 10 °C.min^−1^ from 30 °C to 130 °C. The thermal history was erased with the first run, and then the second heating scan was used to determine the correct glass transition temperature (*T_g_*) of the starch foam composites.

Thermogravimetric Analysis (TGA) was performed on TGA/DSC3+ by METTLER TOLEDO (Bangkok, Thailand). Weight loss of starch foam composite was measured under nitrogen atmosphere with a starting weight of about 10 mg. The samples of thermogravimetric analysis were gradually heated from room temperature to 600 °C with a heating rate of 10 °C.min^−1^.

## 3. Results

### 3.1. Characteristic of Modified Used Palm Oil (mUPO)

Table 2 summarizes the parameters of UPO and mUPO prior to the preparation of the PU adhesive. After converting UPO to mUPO, the iodine number, which indicates the amount of double bonds on the oil structure, reduced from 40.1 to 0.51. The OH values, which indicate the presence of a hydroxyl group on the structure of UPO and mUPO, rose from 0 to 192.19 mgKOH/g. The acid number for free acid in UPO and residual acid after the reaction in MUPO rose slightly from 1.41 to 1.76 mgKOH/g. These findings suggested that UPO is effectively changed by oxidation and hydroxylation to mUPO, which corresponded to the previous works [27,28,29].

**Figure 1:**
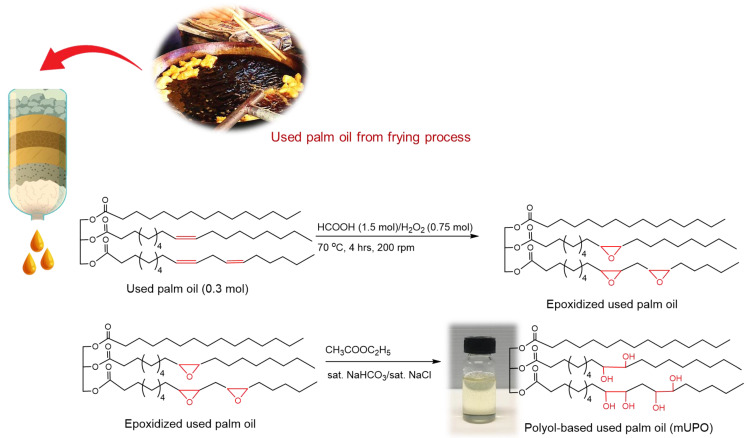
The modification process and chemical structure of UPO and mUPO.

Additionally, the molecular weight of UPO and mUPO is determined using the SEC method. Because of the breakage of a double bond to create OH functional groups on the mUPO structure, as shown in Figure 1, the molecular weight value rises from 2841 to 3073 g/mol. The PDI has fallen from 1.08 to 1.02.

### 3.2. Characteristic of Non-Glutinous Thermoplastic Starch Foam

#### 3.2.1. The Shrinkage of Non-Glutinous Thermoplastic Starch Foam

In order to gain the production for the industrial sector, the shrinkage of the starch foam is initially assessed.

**Figure 2:**
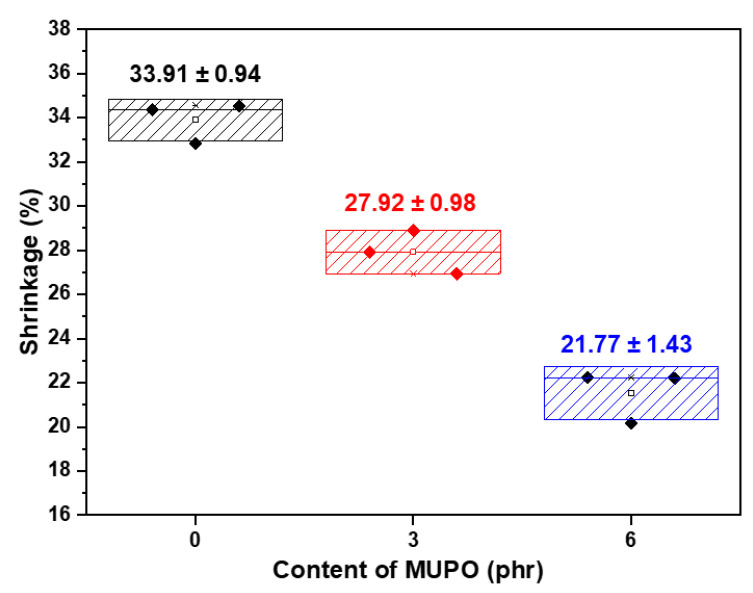
The shrinkage percentage of non-glutinous starch foam with various mUPO content—0, 3, and 6 phr.

Figure 2 displays the proportion of shrinking as a result. By adding mUPO at levels of 0, 3, and 6 phr, the shrinkage results of the foam composite are 33.91, 27.92, and 21.77%, respectively. The findings support the work of Hadi and Babaluo [32], who utilized dioctylphthalate (DOP) as a plasticizer of polyvinyl chloride (PVC) sheet to lessen the shrinkage of the sample. According to reports, adding plasticizer reduced the product shrinkage from approximately 13.4% to 9.8% by raising the DOP content from 35 to 45 wt%. The purpose and effects of plasticizers can be used to describe it. Plasticizers typically have low molecular weight and spread polymer chains apart by forming secondary bonds with them. Therefore, plasticizers increase chain mobility and decrease secondary polymer–polymer bonding, resulting in a softer mass and lower modulus materials [18]. In this study, the primary chain structure of the starch was affected by a large number of hydroxyl groups on the mUPO molecule. As a result, mUPO decreased starch chain entanglements, resulting in less shrinkage.

#### 3.2.2. The Moisture Absorption of Non-Glutinous Thermoplastic Starch Foam

**Figure 3:**
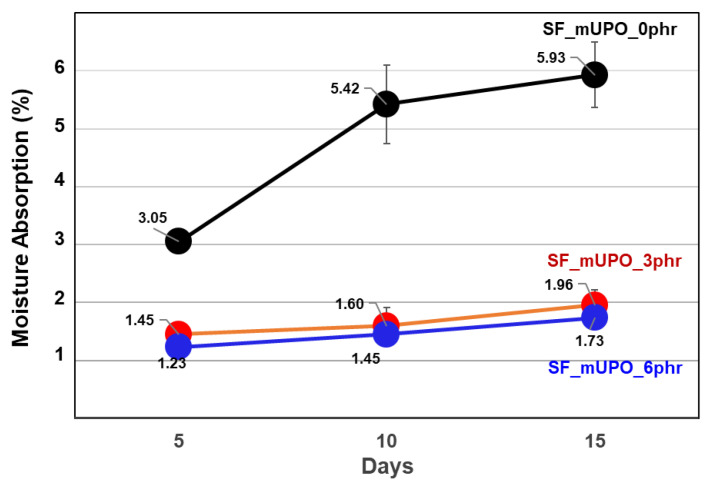
The moisture absorption at 70% relative humidity of non-glutinous starch foam with various mUPO content—0, 3, and 6 phr.

Moisture absorption at 70% relative humidity is another form of property testing to access the properties while in use. Figure 3 illustrates the results of a 15-day collection and monitoring of input. Moisture absorption without mUPO was 3.05, 5.42, and 5.93% after 5, 10, and 15 days, respectively. The moisture absorption results at day 5, 10, and 15 after 3 phr of mUPO addition into the system of non-glutinous starch foam were 1.45, 1.60, and 1.96%, respectively, whereas after 6 phr of MUPO addition, they were 1.23, 1.45, and 1.73%, respectively. The collected data reveals that the addition of MUPO plasticizer can significantly reduce moisture absorption and prolong the life of starch foam by preventing fungi growth. The effects of low hydrophilic agent addition into the high hydrophilic matrix can be used to describe the reduction in moisture absorption. Because of the triglyceride structure of fatty acids, mUPO with 192 potions of hydroxyl group are normally low hydrophilic molecules. When high molecular weight and hydrophilic molecules, mUPO, are inserted to the starch matrix, the hydrophilicity of starch foam is decreased.

It is because of the hydrophilic positions, along with hydroxyl groups and ether groups of starch, that the hydrogen bond with the hydroxyl group formed about 192 positions per chain of mUPO. Accordingly revealing, only the hydrophobic backbone and covering in the surface of starch foam are found. Moreover, the findings are related to the work of Nykänen et al. [20]. The water absorption of the starch foam was reduced from 11 to 9.2% by adding 0 to 10 wt% of hydrophobic plasticizer. Another study by Andreuccetti et al. [33] reported on the incorporation of a hydrophobic plasticizer (acetyltributyl citrate and tributyl citrate) into a gelatin-based film system. The addition of a hydrophobic plasticizer could reduce water permeation. Therefore, in this study, the addition of low hydrophilic mUPO to non-glutinous starch foam can reduce the moisture absorption percentage as well.

#### 3.2.3. The Mechanical Properties of Non-Glutinous Thermoplastic Starch Foam

##### Impact Strength

One of the most common functions of a plasticizer is to aid the improvement of polymer properties, particularly mechanical properties. In the industrial sector, when incorporated into a plastic, elastomer, or even thermoplastic starch, they help improve the properties of flexibility, extensibility, and processibility [34].

**Figure 4:**
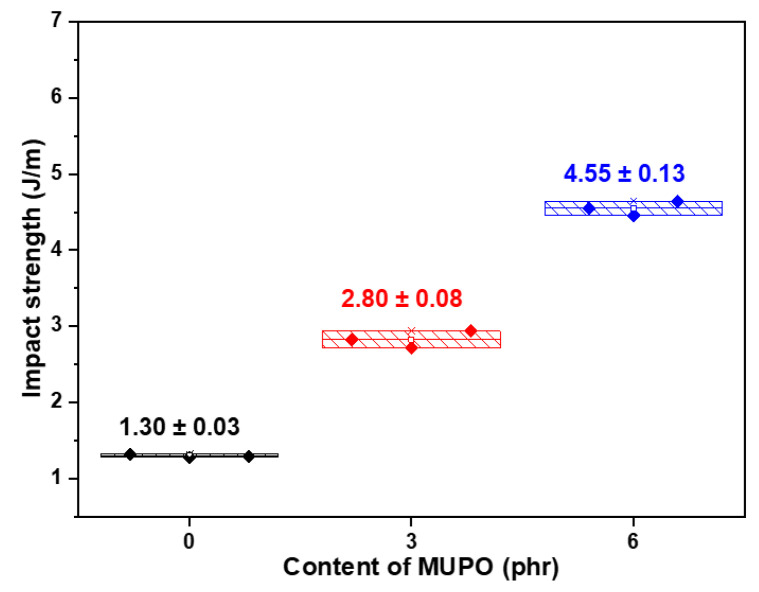
The impact strength of non-glutinous starch foam with various mUPO content—0, 3 and 6 phr.

The mechanical properties of starch foam are comprehensively evaluated in this work to assess the aforementioned characteristics, utilizing flexural and impact tests to assess the role of mUPO as a plasticizer. Firstly, the impact strength of the samples is studied and reported. Figure 4 shows the impact strength of non-glutinous starch foam with varying mUPO content. The result indicates that increasing the mUPO content increased the impact strength by 1.30, 2.80, and 4.55 J/m by adding the mUPO of 0, 3, and 6 phr, respectively. The addition of 6 phr of mUPO increases the impact strength of starch foam by threefold. These findings are related to the research of Kormin et al. [35], who investigate how plasticizers affect the characteristics of starch/low density polyethylene (Starch/LDPE). The findings demonstrated that adding plasticizer increased impact strength, and that this gain sustained as plasticizer content increased. Tarique et al. [23] investigated the effects of glycerol plasticizer on the properties of arrowroot (*Maranta arundinacea*) starch biopolymers. The brittleness and fragility of the starch film could be reduced by adding glycerol. In comparison, pristine plasticizers used as much as 5–40%, while MPUO plasticizer in this work can be used at only 6 phr as an effective plasticizer. Therefore, it should be noted that this is the first evidence that mUPO could plasticize starch foam by tripling loading efficiency compared to pristine starch foam.

##### Flexural Test

**Figure 5:**
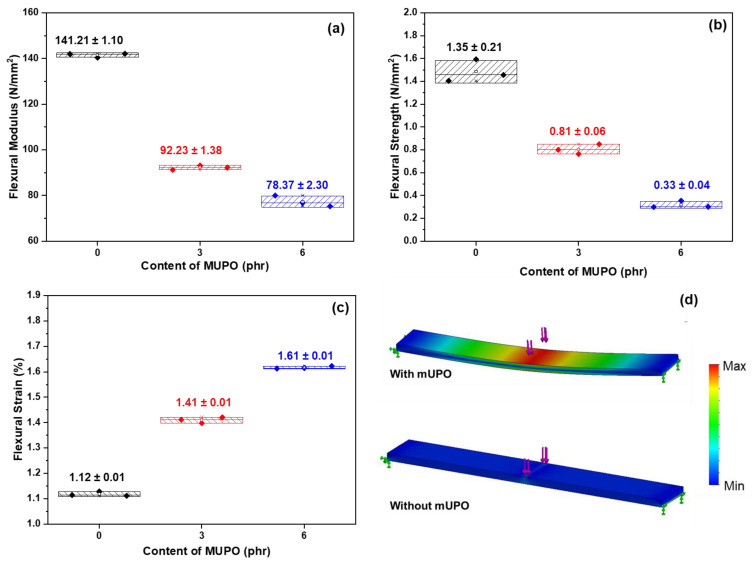
The results of (**a**) flexural modulus, (**b**) flexural strength, (**c**) flexural strain, and (**d**) simulation of non-glutinous starch foam with various mUPO content—0, 3, and 6 phr.

A three-point bending flexural test is also used to evaluate the mechanical properties of starch foam composite. In this work, a flexural test is used to determine the flexibility of a non-glutinous starch foam by measuring the force required to bend a beam while loaded. There are three types of data in flexural test reports: flexural modulus, flexural strength, and flexural strain. Figure 5 illustrates the flexural properties of non-glutinous starch foam when the mUPO-based plasticizer was varied by 0, 3, and 6 phr. The flexural modulus and flexural strength tendencies are the same as shown in Figure 5a,b. Increased mUPO content decreased flexural modulus and strength.

The flexural modulus of non-glutinous starch foam with mUPO contents of 0, 3, and 6 phr is 141.21, 92.23, and 78.37 N/mm^2^, respectively. Similarly, the flexural strength of starch foam with varying mUPO content of 0, 3, and 6 phr is 1.35, 0.81, and 0.33 N/mm^2^. The flexural modulus, also known as the bending modulus, is an intensive property in mechanics that is measured as the stress-to-strain ratio in flexural deformation, or the tendency of a material to resist bending [36]. The stress-to-strain ratio in flexural deformation is reduced as a result of the mUPO-based plasticizer. Flexural strength, on the other hand, is defined as the stress in a material just before it yields in a flexural test. Thus, the flexural strength represents the maximum stress experienced within the material at the moment of yield [37].

Flexural strain is the last flexural result to access mechanical properties. The nominal fractional change is in the length of a test specimen element of the outer surface at midspan, where the maximum strain occurs. As a result, the result can refer to the distance-change percentage of the stretched sample. Figure 5c illustrates the flexural strain of starch foam as the MUPO-based plasticizer content is increased. It is discovered that increasing the mUPO from 0 to 3 phr can increase the flexural strain from 1.12, 1.41, to 1.61%. According to the results of the flexural test, a mUPO-based plasticizer can facilitate chain mobility more than a pristine one. It is related to the work of Watkinson and Mohsen [38], who investigated the effects of plasticizer type and content on polyvinyl chloride properties. It has been reported that as plasticizer content increases, flexural modulus and strength decrease while flexural strain increases. Schmitt et al. [39] used glycerol and sorbitol as a plasticizer of starch foam in a similar study. After incorporating the plasticizer, the foam became softer and its mechanical properties decreased. The effects of a mUPO-based plasticizer can be described based on the flexural study. Plasticizers, as previously stated, spread polymer chains apart by forming secondary bonds with them [18]. The results show that mUPO-based plasticizers increase chain mobility while decreasing secondary polymer–polymer bonding, resulting in a softer mass and lower modulus materials. The plasticizer improves thermoplastic starch displacement, as confirmed by Özeren et al. [40]. Therefore, the strain of thermoplastic starch is enhanced by adding a mUPO-based plasticizer.

The simulation of starch foam composite based on thermoplastic non-glutinous starch is shown in Figure 5d, which is based on the results of mechanical properties: the impact strength and flexural test. In comparison, the load efficiency of the starch foam composite without the mUPO-based plasticizer is lower than that of the mixing one. While the starch foam softens with the addition of a mUPO-based plasticizer, the point of failure demonstrates that it appears to take more force to deconstruct the starch foam than the pristine one.

#### 3.2.4. The Morphology of Non-Glutinous Thermoplastic Starch Foam

**Figure 6:**
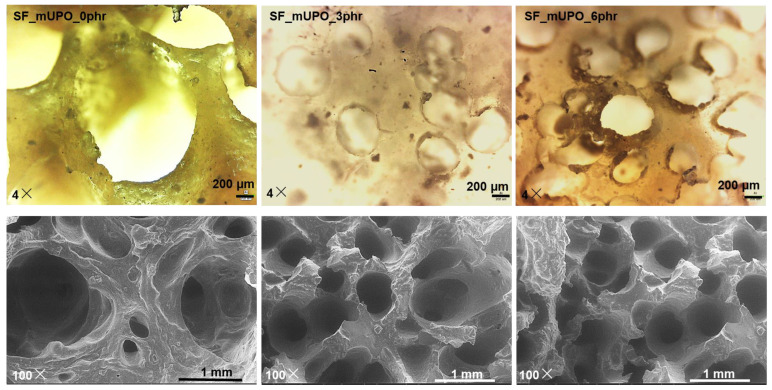
Optical microscope images with a magnification of 4× (upper) and SEM images with a magnification of 100× (lower) of non-glutinous starch foam with various mUPO content—0, 3, and 6 phr.

The morphology of the cross-section of the starch foam is determined using an optical microscope (OM) with a magnification of 4× and a scanning electron microscope (SEM) with a magnification of 100×, as demonstrated in Figure 6. The cross-section of the sample after flexural testing is measured. It is very clear that the mUPO-based polyol can be used as an effective plasticizer for the starch foam composite. The results show that increasing the mUPO content reduces the pore size of starch foam. The addition of a mUPO-based plasticizer can also improve pore size and pore size distribution. The reduction of pore size was observed from about 1.3 mm to 0.3 mm by increasing the mUPO content from 0 to 6 phr. It can be described by the effect and action of the plasticizer of mUPO, as described in the previous section. Because of the lower chain mobility, the starch matrix without a mUPO-based plasticizer has a large cell size. Meanwhile, the starch matrix with mUPO can be moved more easily, resulting in smaller cell size. This is how it can be described. The gas can be fused and pushed into the starch matrix more easily during the foaming reaction than without one. As a result, the cell size and size distribution of the mUPO-treated starch foam are smaller and more homogeneous.

Furthermore, the morphology of starch foam with varying mUPO content can be used to describe the behavior of the starch foam fraction during testing. The addition of mUPO increases chain mobility and load efficiency. Accordingly, in the absence of mUPO, the starch fractures like a brittle material with a smooth surface failure during the progress. The starch foam with mUPO, on the other hand, has a high load resulting from impact strength and flexural findings because the non-glutinous matrix is softer and more flexible. In comparison to plain non-glutinous starch foam, the matrix is stretched like a ductile material during the fraction of the starch foam. These results are related to the work of Yin et al. [41], who studied the effect of ethylene glycol plasticizer on the morphology of PVOH foam. It could affect the foaming process and the cell morphology by decreasing the cell size and melt strength. Another work is by Kahvand and Fasihi [22], who used glycerol and citric acid as a plasticizer of corn starch foam/PVOH, and obtained results confirming that the increase of glycerol and citric acid plasticizer content can improve the distribution and decrease the cell size.

#### 3.2.5. The Thermal Properties of the Thermoplastic Starch Foam

The thermal properties of the starch foam were determined by two techniques: differential scanning calorimetry (DSC) and thermogravimetric analysis (TGA). The results are described regarding the effects of a mUPO-based plasticizer on the thermal properties of starch foam. The thermal stability and transition temperature are presented and discussed. The summary results of thermal properties are listed in Table 3. The glass transition temperature (*T_g_*) of non-glutinous starch foam was measured using DSC analysis, which played an important role in the thermophysical transition. The value of *T_g_* of thermoplastic starch, as defined by Zhang et al. [42], is the temperature at which bounded amylose and amylopectin become loose enough to cause significant movement of the starch molecules.

##### DSC Analysis

**Figure 7:**
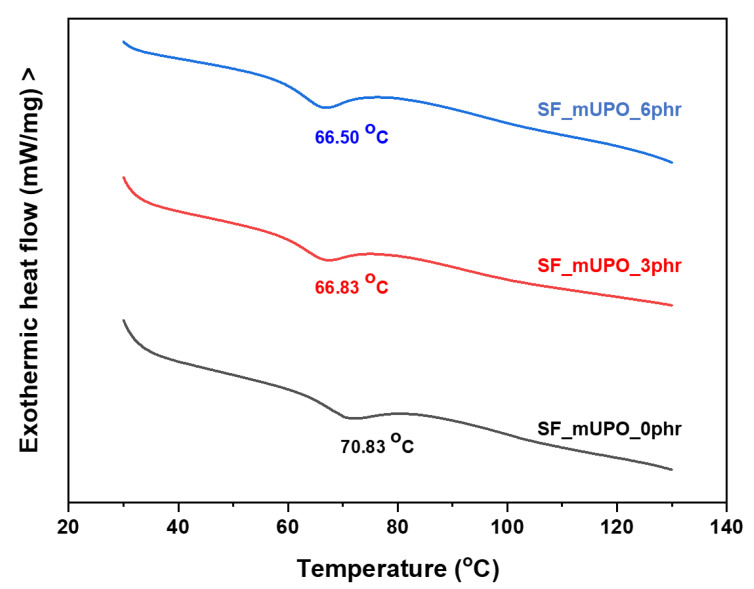
DSC thermogram of non-glutinous starch foam with various mUPO content—0, 3, and 6 phr.

The DSC thermogram of non-glutinous starch foam with various mUPO contents is shown in Figure 7. The addition of the mUPO-based plasticizer from 0 to 3 and 6 phr reduces the *T_g_* values of the starch foam from 70.83, 66.83, and 66.50 °C, respectively. As a result, the *T_g_* values of the non-glutinous starch foam decreased significantly as the mUPO content increased. In comparison, by adding only 3 phr of the mUPO-based plasticizer, the *T_g_* was reduced by about 4 degrees Celsius. Because MUPO-based plasticizers increase chain mobility while decreasing secondary polymer–polymer bonding, *T_g_* values are lower. According to Sanyang et al. [21], increasing the glycerol plasticizer content decreased the *T_g_* values of sugar palm thermoplastic starch. Mali et al. [19] investigated the effects of glycerol plasticizer content on the thermal properties, particularly the *T_g_* value, of various starch sources, including corn, cassava, and yam starch. These findings have a consistent pattern. Tarique et al. [23] also confirmed that the effects of glycerol plasticizer, an effective thermoplastic starch plasticizer, decreased the *T_g_* value of arrowroot (*Maranta arundinacea*) starch. According to the researchers, this plasticizer improves the thermoplastic starch chain movement. It has been stated that the high content of hydrophilic plasticizer allowed for more interactions between plasticizer and starch, resulting in an increase in the free volume of the starch foam and a decrease in the *T_g_* value. This study confirms the role of mUPO molecules in spreading starch chains apart by forming secondary bonds as one of the plasticizers to increase free volume, resulting in a decrease in the *T_g_* value.

##### TGA Analysis

**Figure 8:**
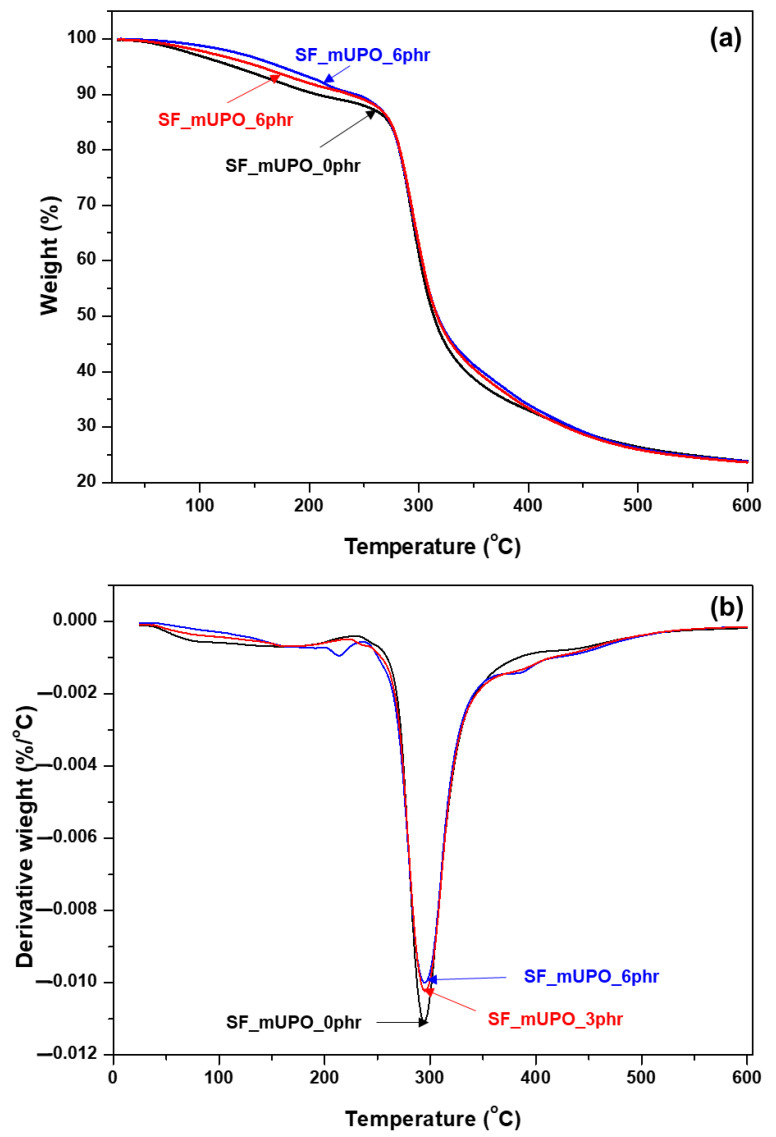
Thermogram of non-glutinous starch foam with various mUPO content— 0, 3, and 6 phr—(**a**) TGA and (**b**) DTG.

The thermal degradation and stability of pristine non-glutinous starch foam and plasticized non-glutinous starch foam were determined using TGA and DTG. The concentration of the mUPO-based plasticizer was varied between 0 and 6 phr. Figure 8 illustrates the TGA and DTG thermograms as a function of temperature (°C) as described by the weight decomposition (%). In the thermal decomposition of the starch foam composite, there are three steps: two for moisture loss and one for main decomposition.

The first step of the thermal decomposition of the starch foam occurred below 100 °C, at around 50 °C, due to moisture loss from the non-glutinous starch foam. Concurrently, this step was linked to mass loss, which can be attributed to the loss of weak bonds between water molecules as well as low molecular weight molecules in the starch foam [23]. Figure 8a,b shows that pristine starch foam decomposed faster than plasticized biopolymers at temperatures below 100 °C. This could be attributed to the fact that the pristine starch foam had a higher water content than the plasticized starch foam, depending on moisture absorption results, which agreed with other reports [21,23].

The second step of thermal decomposition occurred at temperatures ranging from 125 to 280 °C, and it was strongly linked with the break of mUPO and loss of water molecules. These thermal decomposition results agreed well with the findings of Riyapan et al. [30]. Furthermore, the loss of the glycerol plasticizer in the thermoplastic starch is congruent with this decomposition step [19,21,23]. As shown in Figure 8, it is very clear to confirm that the mUPO can improve the thermal decomposition of the non-glutinous starch foam by this step of decomposition.

The loss of hydrogen functional groups, degradation, and depolymerization of the starch carbon chains polymer may be attributed to the final step of decomposition [23]. According to Figure 8 and Table 3, the *T_max_* of the starch foam with mUPO contents of 0, 3, and 6 phr is 294.17, 294.67, and 295.00 °C, respectively. While the percentages of decomposition are 49.03, 48.38, and 48.02%, they decrease as the mUPO content increases. Because of the strong contact between the mUPO-based plasticizer and the non-glutinous starch matrix, the decomposition temperature of the plasticized starch foam is higher than that of the pristine starch foam. While in this state, the mUPO decomposes faster than the pristine starch foam. The mass residual percentage of the starch foam, however, is not significantly different.

The thermal decomposition kinetics of the thermoplastic non-glutinous starch can be used to assess the thermal stability of materials in the step of degradation indicated by TGA and DTG data, ascribed by the activation energy (E_a_) of degradation [43,44,45,46]. It can be calculated via thermogram curves by the Horowitz–Metzger equation as shown in Equation (3):

(3) $$\ln[-\ln(1-\alpha )]=\frac{-E_{a}\theta }{\mathrm{RT}s^{2}}+\;C$$

where: *α* represents the fraction of weight loss during degradation in function of time, *θ* = (T − T_s_), where T_s_ is the temperature corresponding to the peak appeared in the DTG curve, E_a_ is the activation energy of degradation to be calculated, and R is the gas constant (8.314 J K^−1^ mol^−1^). The plot of ln[−ln(1 − *α*)] versus *θ*, in range of the DTG peak, gives a straight line. By the least-square method, the kinetic parameters of thermal degradation can be calculated from the slope of the graph and the intercept of the straight line can also be calculated.

The summary of E_a_ of the degradation of thermoplastic non-glutinous starch is shown in Table 3. It is discovered that increasing the content of mUPO-based plasticizers improves the decomposition kinetic. The E_a_ of the degradation values for thermoplastic non-glutinous starch with mUPO contents of 0, 3, and 6 phr are 0.88, 1.05, and 7.88 kJ/mol, respectively. This study implies that mUPO can also insert and react with the starch chain, thereby improving the thermal stability of thermoplastic starch foam. According to the findings, the addition of 6 phr of mUPO helps increase thermal resistance by tenfold compared to the pristine formulation.

**Figure 9:**
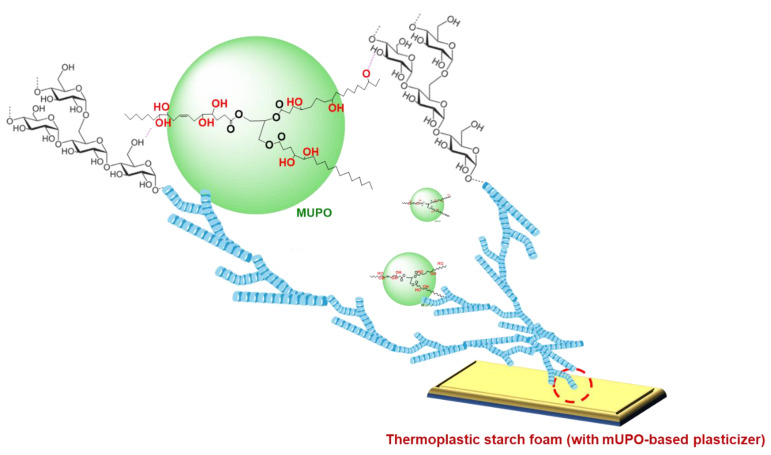
The possibility of the action of the mUPO on the thermoplastic non-glutinous starch foam.

Based on the findings, it can be concluded that mUPO has an effect as a plasticizer of thermoplastic non-glutinous starch foam. The increased flexibility of the thermoplastic starch foam was caused by inserting the molecule of mUPO into the starch foam and its reaction with the starch foam as a secondary bond. The addition of a mUPO-based plasticizer increases the chain mobility and free volume of the thermoplastic non-glutinous starch, resulting in a reduction in shrinkage percentage and moisture absorption, as well as improved impact strength and thermal properties. Furthermore, the mUPO aids in the homogeneous spread of foam formation. Accordingly, the possibility of mUPO action on the thermoplastic non-glutinous starch foam is represented in Figure 9.

## 4. Conclusions

In this study, a waste-based polyol derived from mUPO, with a molecular weight of 3150 g/mol and a hydroxyl group of 192.19 mgKOH/g, has been discovered as an effective plasticizer of starch foam. According to the findings, the optimal mUPO content as an additive was 6 wt%. The addition of mUPO had a direct impact on the mechanical properties of the starch foams, increasing their toughness and impact strength by about threefold. It also improves thermal decomposition and broadens the temperature range. The impact strength increased to 4.55 J/m and *T_g_* dropped to 66.50 °C by adding 6 phr of mUPO in the thermoplastic starch foam. Furthermore, the mUPO could be able to reduce shrinkage to 21.77% and moisture absorption to 1.73%. The morphology study revealed that the mUPO can also reduce the viscosity of the foam. As a consequence, the mUPO derived from waste could be a promising green plasticizer for starch foam applications.

## Figures and Tables

**Table 1:** The formulation of non-glutinous starch foam.

Code Name.	Starch (wt%)	PVOH (wt%)	Baking Powder (phr)	mUPO (phr)
SF_mUPO_0phr	92.5	7.5	7.5	0
SF_mUPO_3phr	92.5	7.5	7.5	3
SF_mUPO_6phr	92.5	7.5	7.5	6
SF_mUPO_9phr *	92.5	7.5	7.5	9

* Note: The formulation of 9 phr MUPO is overused and has bloomed out of the surface. As a result, this formulation has not been characterized.

**Table 2:** Summary of properties of UPO and mUPO.

Code Name	Physical Appearance	Properties	Results
**UPO**	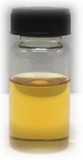	Iodine number	40.1
		OH value (mgKOH/g)	0
		Acid number (mgKOH/g)	1.41
		^a*^ M_n_ (g/mol)	2841
		^a*^ M_w_ (g/mol)	3074
		^a*^ PDI	1.08
**mUPO**	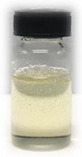	Iodine number	0.51
		OH value (mgKOH/g)	192.19
		Acid number (mgKOH/g)	1.76
		^a*^ M_n_ (g/mol)	3073
		^a*^ M_w_ (g/mol)	3150
		^a*^ PDI	1.02

^a*^ Molecular weight by SEC.

**Table 3:** The thermal property results of non-glutinous starch foam composite.

Code Name	TGA/DTG				DSC
	Main Thermal Decomposition			Residual Mass (%)	*T_g_* (°C)
	*T_max_* (°C)	Decomposition (%)	Ea of Degradation (kJ/mol)		
**SF_mUPO_0phr**	**294.17**	49.03	0.88	23.86	70.83
SF_mUPO_3phr	294.67	48.38	1.05	23.85	66.83
SF_mUPO_6phr	295.00	48.02	7.88	23.65	66.50

## Data Availability

The study did not report any data.

## Supplementary Materials

The following supporting information can be downloaded at: https://www.mdpi.com/article/10.3390/polym14193997/s1, Figure S1: ^1^H-NMR spectra of UPO and mUPO; Figure S2: FTIR spectra of UPO and mUPO.
