# Supplementary figures and images for: The Use of New Waste-Based Plasticizer Made from Modified Used Palm Oil for Non-Glutinous Thermoplastic Starch Foam
Source: Polymers (Basel). 2022 Sep 24;14(19):3997. doi: 10.3390/polym14193997 (PMC9572336; doi:10.3390/polym14193997)

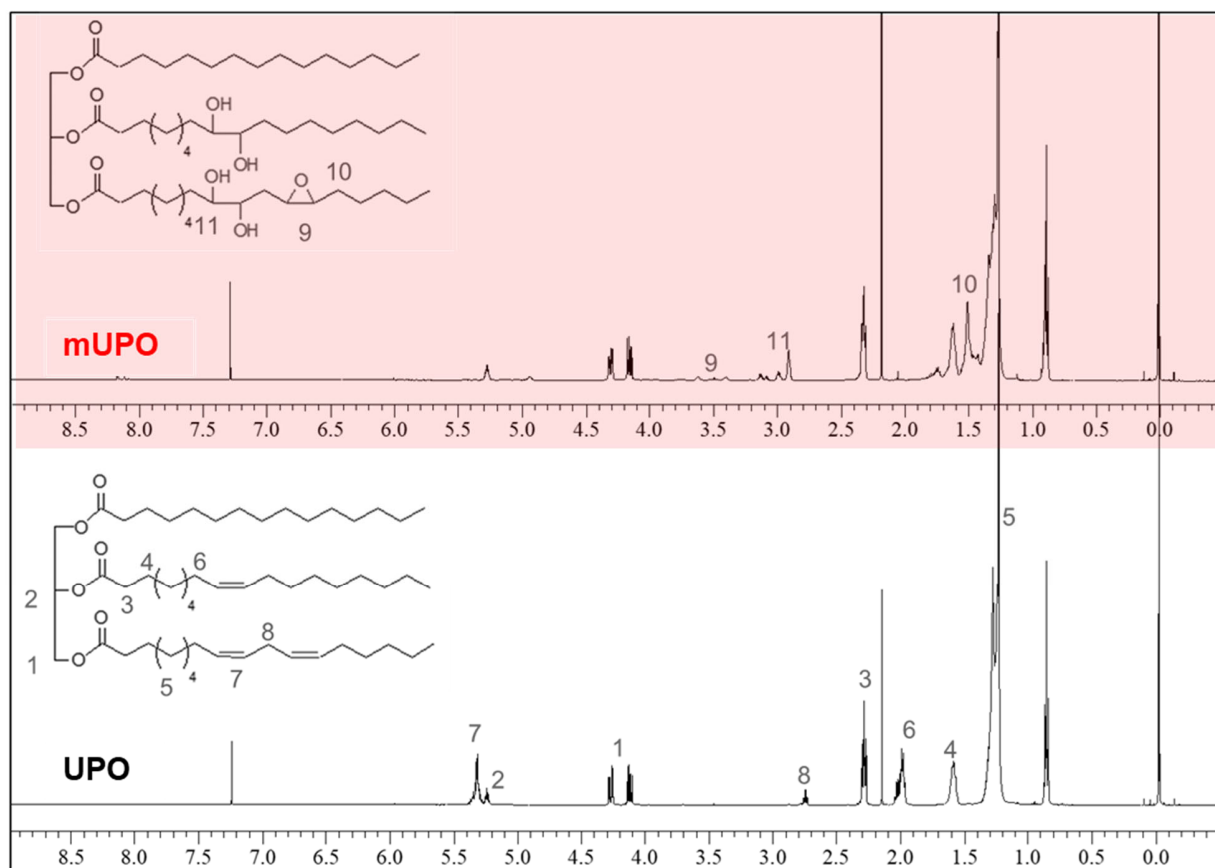

**Figure S1.** <sup>1</sup>H-NMR spectra of UPO and mUPO.

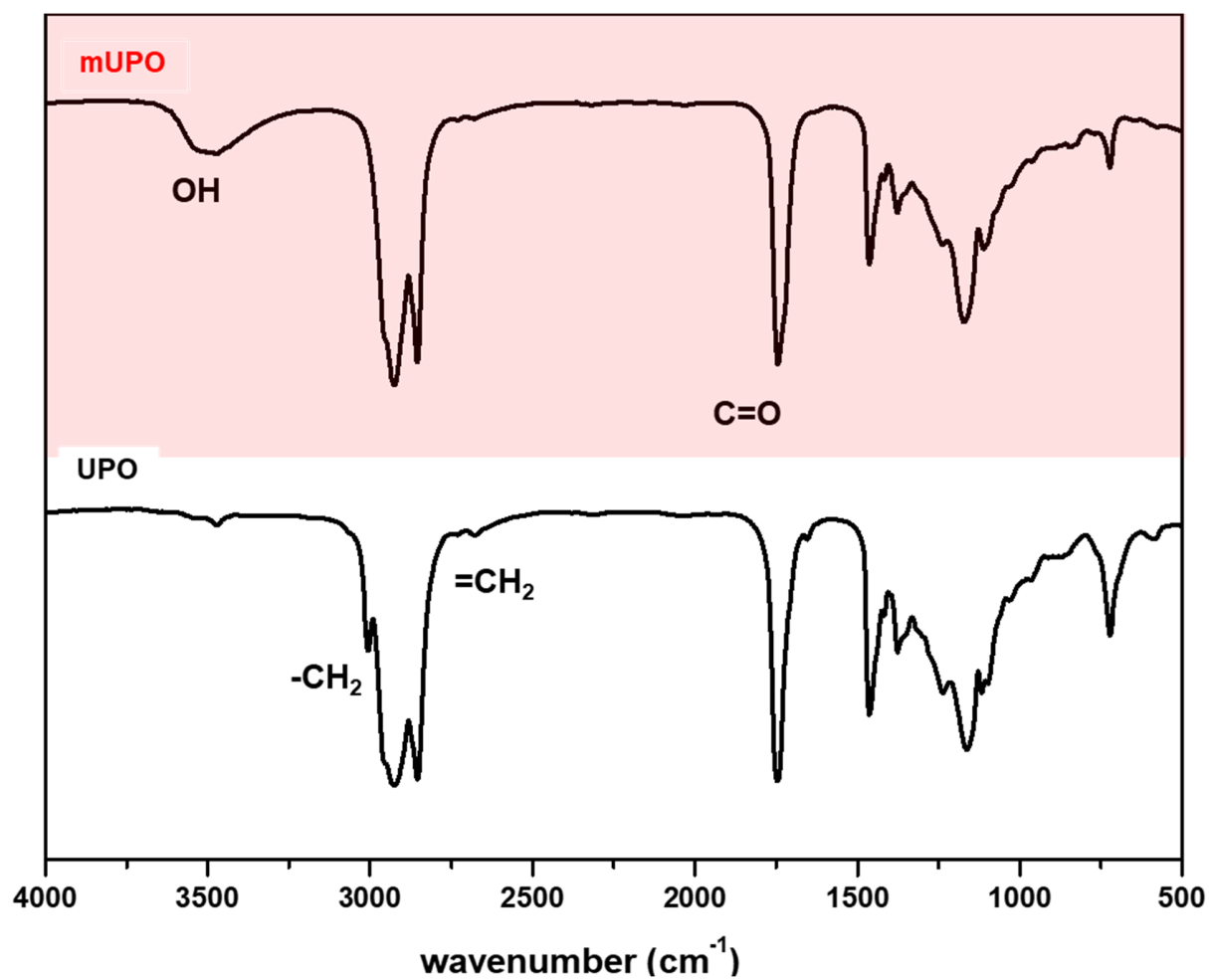

**Figure S2.** FTIR spectra of UPO and mUPO.

Supplement: Supplementary file 1 [file polymers-14-03997-s001.zip › polymers-1927887-supplementary.pdf]
